# Exploring Mixed Ionic–Electronic-Conducting PVA/PEDOT:PSS Hydrogels as Channel Materials for Organic Electrochemical Transistors

**DOI:** 10.3390/polym16111478

**Published:** 2024-05-23

**Authors:** Tatiana Gregorio, Dominique Mombrú, Mariano Romero, Ricardo Faccio, Álvaro W. Mombrú

**Affiliations:** Centro NanoMat & Área Física, Departamento de Experimentación y Teoría de la Estructura de la Materia y sus Aplicaciones (DETEMA), Facultad de Química, Universidad de la República, Montevideo 11800, Uruguay; tgregorio@fq.edu.uy (T.G.); rfaccio@fq.edu.uy (R.F.)

**Keywords:** PEDOT:PSS, hydrogel, channel material, organic electrochemical transistor, organic mixed ionic–electronic conductor

## Abstract

Here, we report the preparation and evaluation of PVA/PEDOT:PSS-conducting hydrogels working as channel materials for OECT applications, focusing on the understanding of their charge transport and transfer properties. Our conducting hydrogels are based on crosslinked PVA with PEDOT:PSS interacting via hydrogen bonding and exhibit an excellent swelling ratio of ~180–200% *w*/*w*. Our electrochemical impedance studies indicate that the charge transport and transfer processes at the channel material based on conducting hydrogels are not trivial compared to conducting polymeric films. The most relevant feature is that the ionic transport through the swollen hydrogel is clearly different from the transport through the solution, and the charge transfer and diffusion processes govern the low-frequency regime. In addition, we have performed in operando Raman spectroscopy analyses in the OECT devices supported by first-principle computational simulations corroborating the doping/de-doping processes under different applied gate voltages. The maximum transconductance (g_m_~1.05 μS) and maximum volumetric capacitance (C*~2.3 F.cm^−3^) values indicate that these conducting hydrogels can be promising candidates as channel materials for OECT devices.

## 1. Introduction

The field of wearable devices that can monitor health from biological fluids such as total analysis systems, wearable sweat-sensing patches, and smart sutures/bandages has grown exponentially in the last decade [1]. The use of conjugated polymers for these applications is especially attractive due to their chemical stability, facile processability, biocompatibility, and electronic and ionic transport, as well as the fact that they enable a compatible interface with living cells and tissues [2,3,4,5,6]. Some of the most popular conducting polymers that have shown excellent properties working as active materials for different types of organic electronic devices such as transistors are those based on polythiophene derivatives [7,8]. Particularly, organic electrochemical transistors (OECTs) are a new class of device that demonstrate some of the many attributes of conjugated polymers such as excellent and tunable mixed ionic–electronic transport properties [8]. Basically, in OECT devices, the ions from an electrolyte are gated and transported to the channel material, which is a conjugated polymer network, and modulate the conductivity through intricate ion–electron coupling [9,10,11,12,13]. OECTs can amplify small chemical signals with high sensitivity and thus are especially promising for diverse biomedical applications such as the detection of ions [14,15] and other metabolites with biological relevance [16,17,18,19,20]. Transconductance (g_m_) is one of the most relevant parameters to characterize the OECT performance and is calculated in aqueous solution compared to unswollen hydrogel, following g_m_ = ∂I_ds_/∂V_g_, with I_ds_ being the channel current and V_g_ the gate voltage. Mixed ionic–electronic-conducting poly(3,4-ethylenedioxythiophene):poly(styrenesulfonate) (PEDOT:PSS) has been thoroughly used as an OECT channel material, exhibiting outstanding channel current and transconductance values (up to ~2 mS) [21]. The use of additives such as ethylene glycol [22] and ionic liquids [23,24,25] has shown an enhancement in the electrical performance of PEDOT:PSS-based channel materials. This is mainly due to their strong molecular interaction with PEDOT:PSS allowing rearrangements for the conducting PEDOT chains that yield effective electronic transport [23,26]. Also, the addition of crosslinkers such as (3-glycidyloxypropyl)trimethoxysilane (GOPS) [27,28,29,30] and divinyl sulfone (DVS) [31,32] has also been studied as a strategy to improve the stability of OECT devices. In addition, there have been efforts to improve the mechanical properties of PEDOT:PSS channel material by using polymeric additives such as polydimethylsiloxane (PDMS) [33] and poly(2-acrylamido-2-methyl-1-propanesulfonic acid) (PAAMPSA) [34], which exhibit excellent properties as OECT channel materials. However, another key aspect to enhance OECT device performance is the swelling ability of channel materials, whose mission is to improve ionic species transport without drastically reducing the electronic transport in the channel. The field of ionic liquid systems has shown great potential in their use as highly flexible and self-healing electronic sensors, suggesting that ionic gel solely composed of ionic liquids and gelators is a better choice to avoid the problem of solvent volatilization, especially in complex environmental conditions [35]. Recent studies also showed that ionogels exhibit fast gelation and high adhesion due to strong intra- and inter-molecular interactions, even in harsh environments [36]. In addition, there have been recent advances in the field of hydrogel electronics based on a curable hydrogel-based supporting matrix and a stretchable silver–hydrogel ink exhibiting excellent mechanical and electrical conduction properties [37]. Quite recently, PVA and gelatin hydrogel additional layers deposited on PEDOT:PSS films were tested as channel materials exhibiting excellent transconductance (up to ~54 mS) and self-healing properties towards OECT channel scratching [38,39,40]. In the case of hydrogels, compared to typical elastomers, their elasticity comes from their solid polymer matrix, while their viscosity originates from their polymer network mobility as well as water and other components comprising the aqueous phase [41]. The additional hydrogel upper layer is a key aspect of improving the mechanical stability of the OECT channel material but also serves as a tunable ionic transporting layer. However, up to now, and to the best of our knowledge, the use of PEDOT:PSS-based mixed ionic–electronic-conducting hydrogels directly as channel materials has been rarely explored. For instance, Kim et al. have shown that PEDOT:PSS crosslinked with polyvinyl alcohol using acid treatment exhibited promising properties as channel materials for OECT devices [42]. They found that increasing the amount of PVA reduced the channel transconductance and volumetric capacitance but also the threshold voltage. They found that the degree of swelling increased with increasing PVA content, but it was not as prominent as that reported in previous studies due to sulfuric acid treatment [42]. There is no clear consensus about the optimal swelling ratio for OECT applications, but the use of mixed ionic–electronic-conducting hydrogels with higher swelling ability directly as channel materials could be very interesting as they can provide a more active interface with the electrolyte and thus be more accessible to charge transfer processes compared to conducting polymeric films or purely ionic-conducting hydrogels. In the present report, we evaluate the preparation and performance of PVA/PEDOT:PSS-conducting hydrogels working as OECT channel materials with a particular focus on shedding some light on their charge transport and transfer properties. Our PVA/PEDOT:PSS-conducting hydrogels were characterized using X-ray diffraction, FT-IR, Raman spectroscopy, scanning electron microscopy, swelling, chronoamperometry, and impedance spectroscopy studies. In addition, we have performed in operando Raman spectroscopy in the OECT devices supported by first-principle computational simulations, evidencing the doping/de-doping processes under different applied gate voltages.

## 2. Materials and Methods

### 2.1. Hydrogels Preparation

First, hydrogels were prepared by dissolving 5.0 g of polyvinyl alcohol (PVA) (purchased from Sigma-Aldrich, St. Louis, MO, USA) with an average molecular weight of 30,000–70,000 in 100 mL of deionized water under vigorous stirring at 60 °C. After that, corresponding amounts of poly(3,4-ethylenedioxythiophene):poly(styrene sulfonate) (PEDOT:PSS) 1.3% *w*/*w* water solution (purchased from Sigma-Aldrich) were added to the PVA solution, under vigorous stirring overnight. Then, HCl was added to correct the pH to ~2 and, subsequently, 0.01 mL of glutaraldehyde (50% aqueous) was added to 20 mL of the previously prepared PVA/PEDOT:PSS solution [43]. The use of acid catalysts is one of the most typical choices for the synthesis of acetals/ketals generated by treating aldehydes/ketones with alcohols [44]. Finally, the solution was poured onto plastic plates and allowed to dry and solidify for 72 h at room temperature. The hydrogels obtained were named PP-0, PP-3, PP–12, and PP-20 referring to 0%, 3%, 12%, and 20% PEDOT:PSS weight fractions, respectively.

### 2.2. Hydrogels Characterization

Hydrogels were studied by X-ray diffraction (XRD) using a Rigaku Miniflex diffractometer (Japan) working in the Bragg Brentano configuration with Cukα radiation in the 2θ = 5–60° range using 2θ steps of 0.02° with 20°/min speed. Infrared spectroscopy (FTIR) measurements were carried out with a Shimadzu IRSpirit FTIR-ATR instrument and confocal Raman spectroscopy with a WITec Alpha 300-RA instrument using an excitation laser of 532 nm wavelength and laser power below 10 mW. The microstructure of hydrogels was also studied by scanning electron microscopy using a JEOL JSM-5900LV microscope. For this purpose, small pieces of hydrogel samples were pre-swollen with water and then sputtered with a thin gold conducting layer. The swelling ratio was calculated as the weight fraction increase after swelling the hydrogels by immersion in water with respect to the unswollen hydrogel, as follows:

Swelling (%) = 100 × (w_swollen_ − w_unswollen_)/w_unswollen_

with w_swollen_ and w_unswollen_ being the weight of swollen and unswollen hydrogels, respectively. The swelling ratio as a function of time for each hydrogel was obtained by determining the w_swollen_ every 60 s until constant values were obtained.

### 2.3. Hydrogel-Based OECT Device

**Figure 1:**
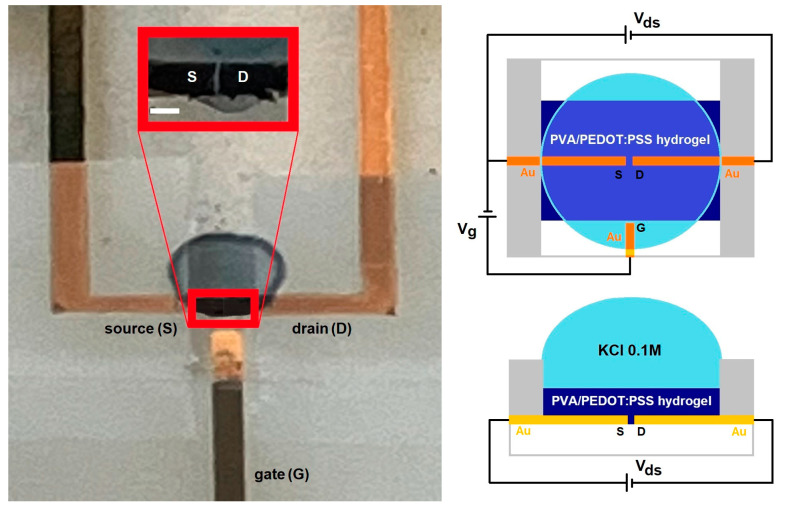
Photo of the OECT device, zooming into the channel area (scale bar 100 μm) (**left panel**), and schematization showing top and front views (**right panel**).

The OECT devices were prepared as follows: with a thin gold (Au) gate, source and drain electrodes were sputtered onto a glass substrate in a 0.2 mbar argon atmosphere at a 30 mA DC sputtering ion current for 300 s using a removable insulating polymer template defining a channel area with length L~20 μm and width W~100 μm. Then, a microliter amount (~6 μL) of PVA/PEDOT:PSS hydrogel solution was deposited on the channel area using a micropipette with 0.2 μL precision and dried at room temperature and 50% relative humidity for 20 h, yielding a thickness d~20 μm. An insulating polymer mask was then deposited, leaving only the channel and gate area exposed to a 0.1 M KCl electrolyte solution, as depicted in the right panel of Figure 1.

The chronoamperometry analysis for our OECT devices was collected between the source and drain electrodes (I_ds_) in the V_ds_ = −0.5 V–−0.1 V drain–source voltage range and different applied gate voltages between the gate and the source electrodes in the V_g_ = −0.3 to +0.5 V range using the Keithley 2450 source-meter. In each case, the gate and source–drain voltages were applied for 5 min before measurement, and then, chronoamperometry was collected for 5 min. Impedance spectroscopy data were collected between the gate and short-circuited source–drain electrodes using a Gamry Reference 3000 galvanostat/potentiostat working with an AC voltage amplitude of V_g,ac_ = 20 mV in the 0.01 Hz–1 MHz frequency range. It is important to mention that the hydrogel-based channel needed to be exposed to the electrolyte solution during the whole set of data collection. The in operando Raman spectroscopy analysis for the OECT devices was collected using the same conditions mentioned above but directly on the PVA/PEDOT:PSS hydrogel channel working at selected gate voltages: V_g_ = −0.6 and +0.6 V.

### 2.4. Computational Simulations of Hydrogel-Based OECT Device

We constructed systems consisting of a 3,4-ethylenedioxythiophene hexamer, a protonated styrene sulfonate dimer, and six H_2_O explicit molecules with the inclusion of K^+^ (to simulate the OECT OFF mode condition, V_g_ > 0), Cl^−^ (to simulate the OECT ON mode condition, V_g_ < 0), and KCl molecules (to simulate the OECT neutral condition, V_g_ = 0), totaling 144 and 145 atoms, respectively. We performed calculations based on DFT [45,46] using Gaussian 16 [47], with the hybrid exchange–correlation potential B3LYP [48,49,50,51] and 6–31 G (d,p) basis set for geometry optimizations.

## 3. Results

**Figure 2:**
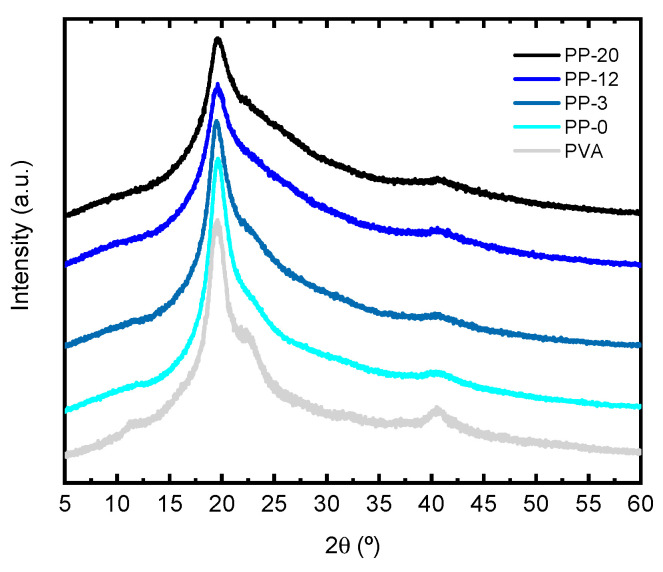
*X*-ray diffraction patterns for PVA, PP-0, PP-3, PP-12, and PP-20 hydrogels.

*X*-ray diffraction patterns for all hydrogels are shown in Figure 2. Pure PVA presents three characteristic peaks in 2θ~20, 23, and 40° ascribed with the (101), (200), and (111) planes of PVA crystallites [52]. The crystallinity index (CI) percentage was calculated by using the following equation:

CI (%) = 100 × (*I_T_* − *I_A_*)/*I_T_*

In the equation, *I_T_* is the intensity of the main peak at ~19.5°, and *I_A_* is the lowest intensity on either side of a crystallinity peak [52]. The crystallinity index (CI) with errors shown in parenthesis for pure PVA is 78(2)%, while for the PP-0, PP-3, PP-12, and PP-20 hydrogels, the CI is 78(2), 79(2), 70(2), and 67(2)%, respectively. This may suggest that there are no drastic changes in the crystallinity index except for relatively high concentrations of PEDOT:PSS (i.e., PP-12 and PP-20 hydrogels). There are no significant changes in the position of the peak associated with the (101) plane of the PVA for any of the samples analyzed. However, there are slight changes in the width of the main diffraction peak denoting that hydrogel samples yielded larger peak widths compared with pure PVA. In addition, it is also observed that the higher the amount of PEDOT:PSS in the hydrogel, the larger the peak width. This suggests that the presence of increasing amounts of PEDOT:PSS favors the amorphization of PVA hydrogels.

**Figure 3:**
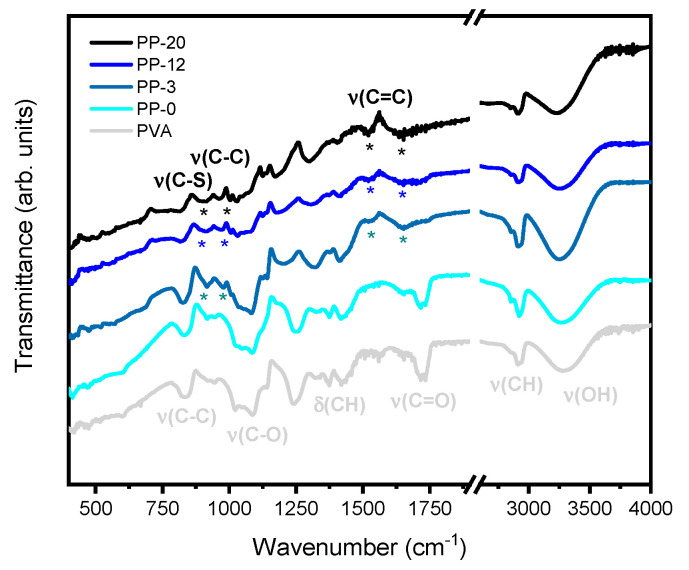
FT-IR spectra for PVA, PP-0, PP-3, PP-12, and PP-20 hydrogels with the asterisks (*) showing the most relevant vibrational modes associated with PEDOT:PSS.

Figure 3 shows the FTIR-ATR spectra for the pure PVA, PP-0, PP-3, PP-12, and PP-20 hydrogels. In all spectra, the characteristic bands of crosslinked PVA like hydroxyl and acetate groups can be evidenced. The broad peak at 3500–3100 cm^−1^ is related to the O-H stretching mode from the intramolecular and intermolecular hydrogen bonds. Interestingly, these peaks exhibit a redshift with increasing amounts of PEDOT:PSS, suggesting that the interaction between each other is probably via hydrogen bonding, thus leading to a decrease in the crosslinking, as already observed in the literature [53]. Furthermore, the peaks around 2840–3000 cm^−1^ can be linked to the C-H stretching modes, and the peak around 1420 cm^−1^ can be associated with the C-H bending modes of PVA. The peaks near 1718 cm^−1^ are probably associated with the C=O stretching modes from partially oxidized PVA but also coexist with those associated with acetal and hemiacetal groups which are products of the crosslinking between PVA and glutaraldehyde [43,54]. Moreover, we can also see evidence of signals corresponding to the structure of PEDOT:PSS such as the characteristic peak at 1647 cm^−1^ attributed to the C=C asymmetric stretching mode for the PEDOT thiophene structure [55,56]. However, it is important to note that these peaks also coexist with those associated with O-H bending modes of PVA. Interestingly, there is a peak at 1525 cm^−1^ which is only present for samples with PEDOT:PSS that can be attributed to the C=C symmetric stretching mode for the PEDOT thiophene structure [56]. The presence of broad peaks at 1090–1030 and 830–840 cm^−1^ can be associated with the C-O and C-C stretching of PVA, respectively, and the peaks at 1250–1200 cm^−1^ can be related to the C-O-C stretching modes of acetal and hemiacetal groups [57].

The narrow peak at 1140 cm^−1^ which is typically related to crystalline regions in PVA is almost absent and vanishes with increasing amounts of PEDOT:PSS [53]. In addition to this, we can find peaks at 976 and 820 cm^−1^ associated with the C-C and C-S stretching mode for the PEDOT thiophene ring, respectively [55].

**Figure 4:**
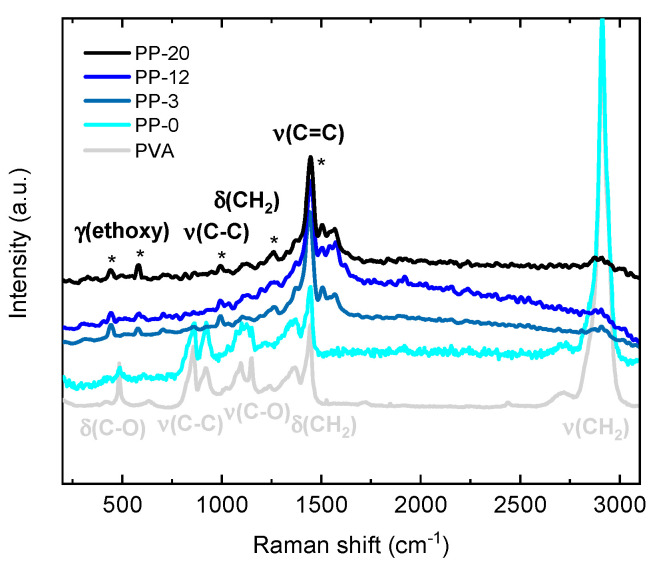
Raman spectra for PVA, PP-0, PP-3, PP-12, and PP-20 hydrogels with the asterisks (*) showing the most relevant vibrational modes associated with PEDOT:PSS.

Figure 4 shows Raman spectra for the pure PVA, PP-0, PP-3, PP-12, and PP-20 hydrogels. The Raman peaks observed at ~417, 485, 631, 857, 918, 1093, 1149, 1369, 1444, and 2910 cm^−1^ can be ascribed to C-O wagging, C-O deformation (δ), O-H wagging, C-C stretching, CH_2_ rocking, C-O stretching, C-O-C stretching, CH_2_ wagging, CH_2_ bending, and CH_2_ symmetric stretching modes of PVA, respectively [58]. PEDOT:PSS typical Raman modes are situated at 430–580, 700, 990, 1100−1135, 1256, 1366, and 1434−1444 cm^−1^ attributed to the ethoxy deformation (γ), C−S−C bending, C−C stretching, C−O−C stretching, CH_2_ wagging, C−C inter-ring stretching, and C=C symmetric stretching of the thiophene rings, respectively [56,59]. Two additional peaks at 1509 and 1567 cm^−1^ are assigned to C=C asymmetrical stretching modes [60,61]. It is important to note that the Raman modes observed for the pure PVA reference material exhibit no drastic modifications for PVA hydrogel samples not containing PEDOT:PSS (i.e., PP-0).

Furthermore, for all the hydrogels containing PEDOT:PSS (i.e., PP-3, PP-12 and PP-20), the peaks assigned to PVA are practically not detected, and only the PEDOT:PSS peaks are observed without drastic differences between each other. This is probably due to the fact PEDOT:PSS exhibits an enhancement in the Raman activity due to resonance conditions.

**Figure 5:**
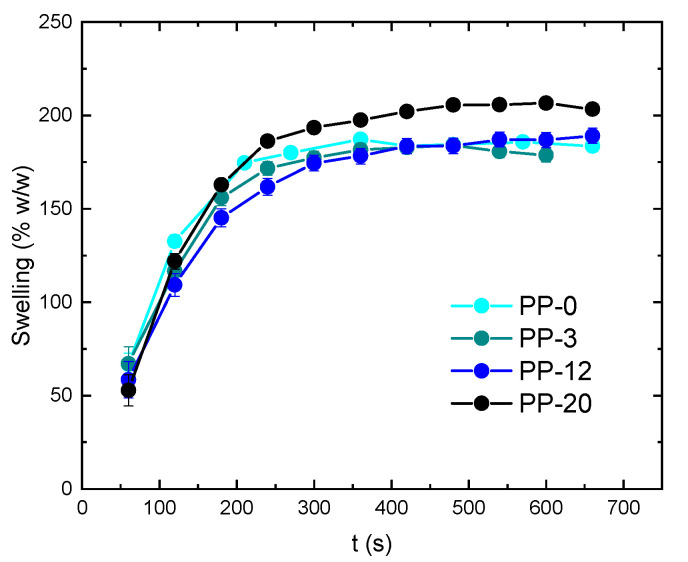
Swelling ratio (expressed in % *w*/*w*) for PP-0, PP-3, PP-12, and PP-20 hydrogels.

The swelling ratio (expressed in % *w*/*w*) for PP-0, PP-3, PP-12 and PP-20 hydrogels is shown in Figure 5. It can be clearly observed that the swelling ratio is almost equal for all cases (~180%), exhibiting a slight increment up to ~200% for PP-20. This suggests that the increment in PEDOT:PSS content does not significantly alter the swelling ability of the hydrogels but, on the other hand, slightly improves it. However, it is important to note that although a slight increment in the swelling is observed for the PP-20 hydrogel, it is also accompanied by a decline in its macroscopical mechanical properties when immersed in the aqueous solution.

**Figure 6:**
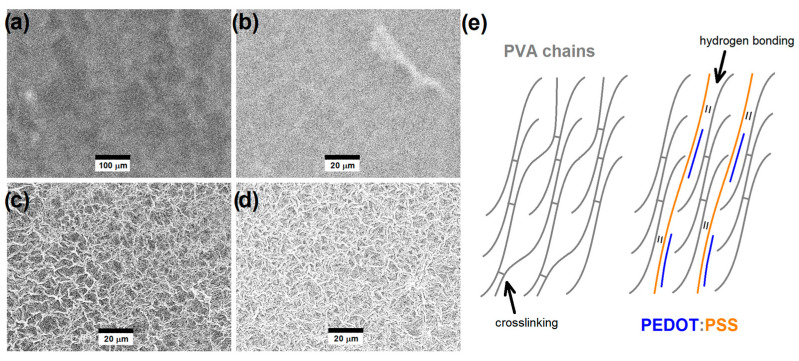
SEM images collected in the secondary electron imaging mode for (**a**) PP-0, (**b**) PP-3, (**c**) PP-12, and (**d**) PP-20 hydrogels after adding a microliter drop of the electrolyte solution. (**e**) Schematization of the PVA interchain interactions via crosslinking and PVA/PEDOT:PSS interchain interactions via hydrogen bonding.

The SEM images collected in the secondary electron imaging mode for previously swollen PP-0, PP-3, PP-12, and PP-20 are shown in Figure 6. The effect of the permeation of the electrolyte solution yields swelling and then the formation of wrinkles in the hydrogels due to the vacuum conditions of SEM instrumentation, particularly evidenced in the presence of higher PEDOT:PSS contents. It is important to note that these are not direct microstructural features of swollen hydrogels, but the comparison between each other allows us to indirectly evaluate the effect of swelling on the hydrogels. The swelling ratio calculated by the swollen/unswollen weight analysis discussed above indicated that there is a slight increase in the swelling ability with the increasing amount of PEDOT:PSS content, particularly for PP-20. The SEM images also suggest that the hydrogels with higher PEDOT:PSS contents, particularly PP-12 and PP-20, are probably forming higher porosity when swollen compared to those with lower PEDOT:PSS contents.

**Figure 7:**
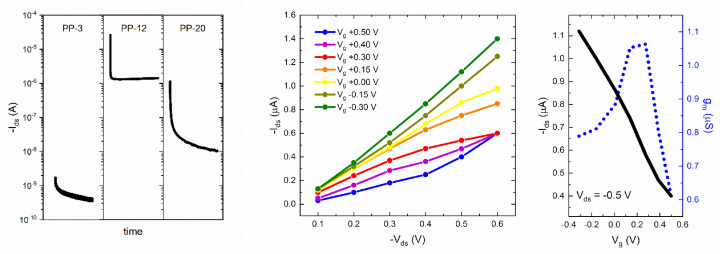
Chronoamperometry at V_ds_ = −0.5 V and V_g_ = −0.3 V for PP-3, PP-12, and PP-20 (**left panel**). Output characteristic at V_g_ = −0.3 V–+0.5 V (**middle panel**) and channel current/transconductance at V_ds_ = −0.5 V (**right panel**) for the PP-12 hydrogel channel OECT devices working with 0.1 M KCl.

The preliminary chronoamperometry studies performed for OECT devices using PP-3, PP-12, and PP-20 showed that the best composition for channel materials was obtained for PP-12, for which acceptable channel currents are observed (I_ds_~−1.1 μA at V_g_ = −0.3 V and V_ds_ = −0.5 V) compared to those using PP-3 (I_ds_~−0.4 nA at V_g_ = −0.3 V and V_ds_ = −0.5 V) and PP-20 (I_ds_ −9.6 nA at V_g_ = −0.3 V and V_ds_ = −0.5 V), as depicted in the left panel of Figure 7. For all cases, the I_ds_ current exponential decay is observed in agreement with the typical in-plane mixed ionic–electronic conduction mechanism of OECT devices, but only PP-12 data exhibit a stable steady-state current.

This probably indicates that the PP-12 hydrogel contains the optimal amount of PEDOT:PSS, enhancing the formation of a continuous electronic-conducting pathway through the hydrogel structure. PP-20 has a higher fraction of PEDOT:PSS-conducting phase, but it can be probably segregated, yielding a global decrease in the continuous electronic-conducting pathway through the hydrogel. Furthermore, the stability of this hydrogel with a higher PEDOT:PSS fraction (PP-20) is considerably lower compared with those with lower PEDOT:PSS contents. For these reasons, further OECT characterization was only performed for the PP-12 hydrogel channel OECT devices. The output characteristic in the V_g_ = −0.3 V–+0.5 V range and the channel current/transconductance at selected V_ds_ = −0.5 V for PP-12 hydrogel channel OECT devices are shown in the middle and right panel of Figure 7, respectively. The output characteristic showed saturation of the I_ds_ current at V_ds_ = −0.5 V for V_g_ = +0.3 V, while saturation was not fully observed for lower and higher gate voltages. Based on the previous feature, we select V_ds_ = −0.5 V to plot the current/transconductance for PP-12 hydrogel channel OECT devices. The application of a positive bias on the gate electrode promotes the K^+^ cations to move into the channel, decreasing I_ds_ in relation to the de-doping process of PEDOT:PSS. The decrease in I_ds_ upon the application of a positive gate bias is consistent with the depletion mode operation, as typically observed in PEDOT:PSS-based OECT devices working with NaCl or KCl electrolytes [21,22,23,24,25,26,27,28,29,30,31,32,33,34,38,39,40,42].

Both I_ds_ and g_m_ values are similar to those reported for OECT devices comprising PEDOT:PSS thin film as channel materials but are considerably lower than those comprising PEDOT:PSS with additives or stabilizers which are typically in the ~mA range [21,22,23,24,25,26,27,28,29,30,31,32,33,34,38,39,40,42]. It is important to mention that we observed a stable current during several cycles (less than 1% variations after 10 cycles). Nonetheless, it is also relevant to mention that the hydrogel-based channel needs to be always exposed to the electrolyte solution during the whole set of data collection and the channel is drastically damaged after drying. The threshold voltage (V_th_) estimated from the I_ds_^1/2^ vs. V_g_ plots was V_th_~0.4 V, which is quite similar to that observed for PEDOT:PSS/PVA hydrogels with lower PVA contents reported by Kim et al. [42]. However, it is important to note that our hydrogel exhibiting the best performance is made of only 12% *w*/*w* of the electronic-conducting polymer PEDOT:PSS and is swollen up to 180% *w*/*w* with the electrolyte solution. In addition, the maximum g_m_ values are observed at V_g_~0.2 V, as similarly observed for PEDOT:PSS thin films with ionic liquid additives [21,22,23,24,25,26,27,28,29,30,31,32,33,34,38,39,40,42]. Impedance spectroscopy experiments were performed to study not only the volumetric capacitance but also the charge transport and transfer mechanism for our conducting hydrogel channel material. The total impedance response can be described by the following equation:

Z = Z′ − *i*Z″

where Z′ is the real part and Z″ the imaginary part of impedance, respectively. The total capacitance as a function of frequency was estimated using the following equation:

C_T_(f) = 1/(2πf|Z’’|)

**Figure 8:**
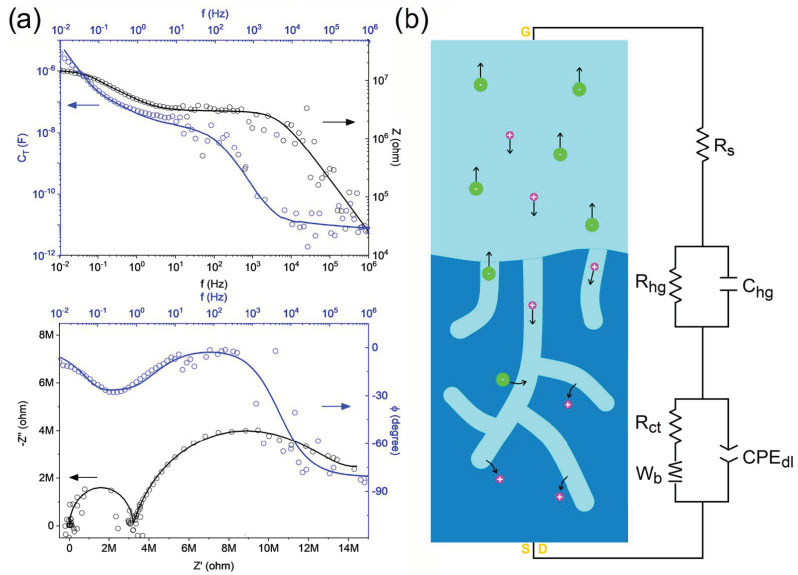
(**a**) Impedance (Z) and total capacitance (C_T_) as a function of frequency and Nyquist/Bode plots obtained from impedance spectroscopy analyses where data is represented with circles and fitting is represented with lines while black and blue arrows indicate the corresponding axis (**b**) Schematization of the circuit model for the ionic–electronic transport for the PVA/PEDOT:PSS (PP-12) hydrogel channel OECT devices working with 0.1 M KCl, where atoms (colors) are potassium (purple) and chloride (green).

The total impedance and capacitance as a function of frequency are shown in Figure 8a. The total capacitance values for the PP-12 hydrogel channel material can be estimated from the plateau at low frequencies, but, in our case, the presence of deviations at low frequencies indicates non-ideal behavior which is more appropriate to study using circuit model fitting [62]. Our impedance data showed best fitting with the circuit model, shown in Figure 8b, composed of a series resistor (R_s_) representing the electrolyte transport in solution, a parallel combination of resistor and capacitor (R_hg_//C_hg_) representing the electrolyte transport through the swollen hydrogel and the parallel combination of resistor, and a bounded Warburg and constant phase element (R_ct_-W_b_//CPE_dl_) representing the electrolyte diffusion and charge transfer processes. The constant phase element (CPE) impedance can be described by the following equation:

Z_CPE_ = (1/Q)/(iω)^α^

where Q (admittance at ω = 1 rad/s) and α (exponent) are frequency-independent parameters, with α = 1 and 0 representing an ideal capacitor and resistor, respectively, while α = 0.5 is associated with diffusion processes [63]. The W_b_ impedance can be described by the following equation:

Z_Wb_ = [(1/Q)/(iω)^1/2^]coth[B(iω)^1/2^]

where B is defined as δ/D^1/2^, with δ being the Nernst diffusion layer thickness and D being the average diffusion coefficient. The fitted R_s_, R_hg_, and R_ct_ values were 500 Ω, 3.2 MΩ, and 10.5 MΩ, respectively, exhibiting a relatively expected trend for resistance values describing the ionic transport in solution, ionic transport in the swollen hydrogel, and charge transfer processes in the polymeric electrode.

The fitted C_hg_ and Q_CPE,dl_ values were 1.0 × 10^−11^ S.s (or F) and 8.4 × 10^−8^ S.s^0.80^, respectively, while the fitted Q_Wb_ and B values were 1.9 × 10^−6^ S.s^0.5^ and 16 s^0.5^, respectively. The Q_CPE,dl_ value was consistent with the C_T_~10^−7^ F total capacitance plateau observed down to ~0.1 Hz depicted in the upper panel of Figure 8a. However, the total capacitance deviation up to C_T_~10^−6^ F observed in the lower frequency regime (~0.1–0.01 Hz) was mostly due to diffusion processes through the conducting hydrogel channel material. Thus, the Q_CPE,dl_ element can be used to describe the non-ideal capacitive process with an associated capacitance C = Q_CPE,dl_(2πf_max_″)^α−1^ = 9.2 × 10^−8^ F, where f_max_″ is the frequency where the imaginary part of impedance has its maximum (~0.1 Hz for this case) [64], and then, the corresponding volumetric capacitance yields C* = 2.3 F.cm^−3^. It is important to mention that this volumetric capacitance value was calculated using L (20 μm) and W (100 μm) determined by the gold drain–source contacts and the thickness of the hydrogels deposited in the OECT channel before being swollen (20 μm), because it is quite challenging to measure the thickness for these thin hydrogel-based channels in the OECT working conditions. However, just to make a speculation on this, the ~180–200% swelling ratio observed for our self-standing hydrogels indicates that all the hydrogels deposited in the channel can almost equally increase their mass (and volume) due to swelling up to a maximum of ~2–3 times without considering that only the upper surface is exposed to the electrolyte solution in the OECT working conditions. Nonetheless, it is relevant to mention that we observed variations in orders of magnitude between I_ds_ for the different hydrogels, and the calculation of the volumetric capacitance was only estimated for the best candidate.

**Figure 9:**
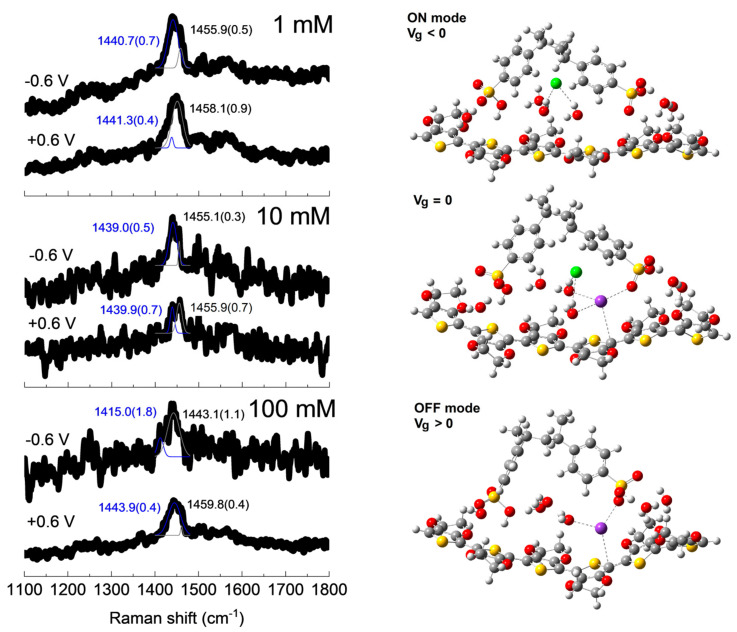
In operando Raman spectra and *v*(C=C) peaks fitting with errors in parenthesis at selected applied gate voltages of V_g_ = −0.6 and +0.6 V for the PVA/PEDOT:PSS (PP-12) hydrogel channel OECT device with KCl solutions with different concentrations (**left panel**) and optimized structures for the modeling of the OECT ON (V_g_ < 0), zero-gated (V_g_ = 0), and OFF (V_g_ > 0) conditions (**right panel**). The lower frequency (1415–1443 cm^−1^) and the higher frequency (1443–1459 cm^−1^) C=C thiophene stretching mode fittings are represented in blue and black, respectively, and references for atoms (colors) are carbon (gray), oxygen (red), hydrogen (white), sulfur (yellow), chloride anion (green), and potassium cation (purple).

To gain more insight into the charge transfer properties, in operando Raman spectroscopy analysis for PVA/PEDOT:PSS hydrogel channel OECT devices at selected gate voltages of V_g_ = −0.6 and +0.6 V were performed, and the results are shown in Figure 9.

It is well known that the peak associated with the symmetric stretching of C=C bonds [*v*(C=C)] at ~1420–1450 cm^−1^ in PEDOT thiophene rings is quite sensitive to the presence of charge carriers due to effective doping [56]. It is interesting to note that there is a blueshift of some of these Raman peaks when the gate voltage varies from −0.6 V to +0.6 V, in agreement with the de-doping of PEDOT:PSS due to the simultaneous removal of chloride anions and introduction of potassium cations into the channel [56]. It is quite challenging to obtain Raman spectra with higher signal-to-noise ratios when collecting data from a working OECT device, mainly due to the presence of the electrolyte solution. In order to have statistical information, we fit our Raman spectra using two peaks (at ~1460–1450 and 1440–1415 cm^−1^), observing, in all cases, a shift toward lower wavenumbers and an increase in the relative intensity of the low wavenumber peak when passing from positive to negative gate voltages, as depicted in the left panel of Figure 9. This also corroborates the depletion mode operation of these PVA/PEDOT:PSS hydrogel channel OECT devices, as already discussed in the OECT device electrical characterization section.

Our first-principle calculations by means of the DFT level of theory indicate that in the ON mode (V_g_ < 0) condition, the chloride anion only interacts directly with surrounding water molecules without altering the doping configuration of PEDOT:PSS. In the zero-gated mode (V_g_ = 0) condition, the presence of the chloride anion is more or less passive, but the potassium cation is more active by directly interacting with thiophene rings of PEDOT but also with the sulfonate group of PSS, as also evidenced for sodium cations using the same level of theory in our recent report [56]. Finally, in the OFF mode (V_g_ > 0) condition, the potassium cation is freer to interact with the thiophene ring of PEDOT and at the same time to block the doping ability of the sulfonate group of PSS, leading to an increment in PEDOT:PSS de-doping.

## 4. Conclusions

Here, we report the preparation and evaluation of PVA/PEDOT:PSS-conducting hydrogels working as channel materials for OECT applications for the first time. In general terms, the hydrogels exhibited a slight decrease in the crystallinity and an increase in the swelling ratio up to ~200% *w*/*w* with increasing PEDOT:PSS content up to 20%. The optimal hydrogel composition for OECT channel material exhibiting better current and transconductance was observed for PVA/PEDOT:PSS with 12% PEDOT:PSS content. The most relevant feature is that the ionic transport through the swollen hydrogel is clearly distinct from the transport in the solution and the charge transfer and diffusion processes govern the low-frequency regime. The in operando Raman spectroscopy analyses directly on the OECT devices supported by DFT calculations showed the doping/de-doping processes under applied gate voltages in correlation with the OECT depletion operation mode. The maximum transconductance (g_m_ ~1.05 μS) and maximum volumetric capacitance (C* ~2.3 F.cm^−3^) values indicate that these conducting hydrogels can be promising candidates as channel materials for OECT devices but, due to their complex ionic–electronic transport/transfer mechanism, a lot of parameters still need to be further studied.

## Data Availability

The data presented in this study are available on request from the corresponding author due to privacy.
